# Priorities Towards Fair Allocation of Ventilators During COVID-19 Pandemic: A Delphi Study

**DOI:** 10.3389/fmed.2021.769508

**Published:** 2022-01-24

**Authors:** Seyyed-Hadi Ghamari, Mohsen Abbasi-Kangevari, Nasim Zamani, Hossein Hassanian-Moghaddam, Ali-Asghar Kolahi

**Affiliations:** Social Determinants of Health Research Center, Shahid Beheshti University of Medical Sciences, Tehran, Iran

**Keywords:** coronavirus infections, health care rationing, ethics, health policy, resource allocation, SARS-CoV-2, mechanical ventilators

## Abstract

**Background:**

COVID-19 pandemic has resulted in severe shortage in vital resources, including invasive mechanical ventilators. The current imbalance between demand and supply of mechanical ventilators has called for investigations on the fair allocation of mechanical ventilators.

**Objective:**

To determine the priorities of the medical experts towards the fair allocation of ventilators during the COVID-19 pandemic.

**Methods:**

This study was conducted from May 28 to Aug 20, 2020. The questionnaire was sent to 50 medical specialists as the Delphi panel. Participants were asked to rate each prioritising factor: “−1” for low priority, “+1” for high priority, and “Zero” for equal priority.

**Results:**

Among 38 experts who responded to the email, the responses of 35 were analysed. 31 (88.6%) participants recommended that pregnant women be considered high priority in allocating ventilators, 27 (77.1%) mothers of children <5 years, 26 (74.3%) patients under 80-years, and 23 (65.7%) front-line-healthcare-workers. In contrast, 28 (80.0) participants recommended that patients who are terminally ill should be considered as a low priority, 27 (77.1%) patients with active-malignancy, 25 (71.4%) neurodegenerative diseases, and 16 (45.7%) patients aged >80. The panel did not reach a consensus regarding the role of patients' laboratory profiles, underlying diseases, or drug abuse in the prioritisation of ventilators.

**Conclusions:**

The panel considered pregnant mothers, mothers of children under 5 years, age groups younger than 80, and front-line healthcare workers to have high priority in allocating mechanical ventilators.

## Introduction

The coronavirus disease 2019 (COVID-19) pandemic is rapidly intensifying worldwide and continues to place an extraordinary burden on humankind (1). Since the early days of the pandemic, a severe shortage in vital resources, including invasive mechanical ventilators, has remained a significant concern of healthcare professionals (2, 3).

During the pandemic, of all patients diagnosed with COVID-19, 17–35% required hospitalisation at ICUs (4, 5) and 9–19% required invasive mechanical ventilation (4, 6). The estimated number of invasive mechanical ventilators in various countries would not be adequate to serve all clinically eligible patients during the current pandemic (3). Therefore, the current imbalance between demand and supply of mechanical ventilators has called for investigations on the fair allocation of mechanical ventilators. Although the research has been ongoing on the subject since the early days of the pandemic, significant concerns remain controversial (7–9).

Medical experts working at the COVID-19 care units interact with patients of different socioeconomic, clinical, paraclinical, and overall health statuses. Nevertheless, physicians should not be faced with situations where they would be obliged to decide which patient to treat due to the risk of human error and the life-long emotional toll (10). Therefore, prioritisation recommendations and guidelines are being developed in the hope of helping physicians, especially those less experienced, with the real-time decision-making process based on the resources and contexts (11, 12). Nevertheless, most studies on the subject have focused on experts' opinions from a single country or region, limiting their generalizability (7–9).

The objective of this study was to determine the priorities of the medical experts towards the fair allocation of ventilators during the COVID-19 pandemic via an international online Delphi survey.

## Materials and Methods

This online Delphi survey has been approved by the Ethical Committee of Shahid Beheshti University of Medical Sciences, Tehran, Iran, under the reference code IR.SBMU.RETECH.REC.1399.103. Participation was anonymous and upon the participant's own decision.

### Review of Literature and Expert Selection

This study was conducted from May 28 to Aug 20, 2020. To design the Delphi questionnaire, an extensive literature review was conducted by the authors. The explored resources for data collection included the Centres for Disease Control (CDC), World Health Organisation (WHO), Infectious Diseases Society of America (IDSA), and European Centre for Disease Prevention and Control (ECDC). Electronic databases including PubMed, EMBASE, Medline, and Cochrane library were precisely investigated using the terms: COVID-19, SARS-CoV-2, mechanical ventilation, prioritisation, healthcare rationing, ethics, health policy, resource allocation, and invasive mechanical ventilators. After the initial preparation of the questionnaire, the Delphi survey was conducted in two phases.

Firstly, an expert panel of 10 individuals, two public health experts, two anesthesiologists, two emergency medicine specialists, two pulmonologists, and two infectious diseases specialists were asked to evaluate the questionnaire and provide other potential variables. The invitation link to participate was sent via email with a brief description of the aim of the study. Data were gathered through an online questionnaire via the Google Form platform.

**Figure 1:**
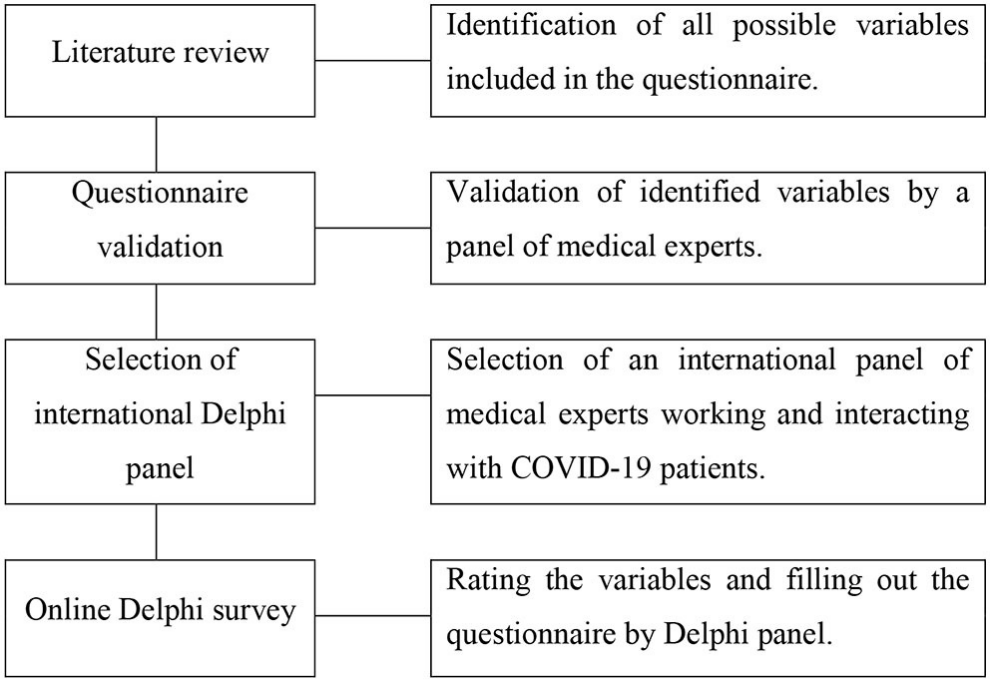
Flowchart of Delphi survey.

Secondly, the revised version of the questionnaire based on the comments of the first-phase panellists was sent to a group of 50 medical specialists as our Delphi panel. All potential members were professionally involved in managing the patients with COVID-19 during the pandemic and were identified by investigating their professional academic curriculum vitae and the acquaintance of authors with them. Of them, 20 were intensive care experts, 10 were internal medicine specialists, 10 were emergency medicine specialists, 5 were forensic medicine experts, and 5 were infectious diseases specialists worldwide. Like the first phase, an email explaining the objective of the survey, their involvement in the study, how the Delphi study works, and the invitation link was sent to each potential participant. Among all participants who received the invitation email, 38 (76% response rate) agreed to participate in the study. Responses of three participants were incomplete, and therefore responses of 35 participants were analysed. Participants' responses in the second phase were considered as final responses. The authors of the article and the medical experts who contributed in the first phase were not included in the Delphi panel (Figure 1).

### Variables and the Questionnaire

Variables included the personal information of the participants and the criteria for prioritising the ventilators during the pandemic. Personal information included participants' gender, age, area of study or speciality, affiliated institution, country, and the ventilator allocation experience in a setting of scarcity during the COVID-19 pandemic.

Among the criteria of prioritising the mechanical ventilators during the pandemic, all potential factors associated with the poor outcome of COVID-19 and the social responsibility of the patient were included and being divided into four sections: (1) non-medical determinants, (2) the underlying health conditions, (3) clinical, and (4) paraclinical presentation of COVID-19. It is worth mentioning that gender differences, religious beliefs, various nationalities, chronic disabilities, and being refugees or immigrants were not included among the criteria of prioritising the mechanical ventilators due to ethical considerations (12).

Among non-medical determinants, various age groups of the patients, being healthcare professionals, smoking status, drug abuse, and being the mother of a child under 5 years were included. Giving the significance of various age groups in COVID-19 prognosis, age groups of patients were considered in eight different groups including below 19, 20 to 49 years, 50–59 years, 60–69 years, 70–74 years, 75–79 years, 80–84 years and above 85 years (13).

The underlying health conditions section included 12 health conditions, which would exacerbate the COVID-19 status of the patient. Obesity, pregnancy, uncontrolled hypertension, ischemic heart disease, poorly controlled diabetes mellitus, chronic kidney disease, neurodegenerative diseases, chronic respiratory failure, organ transplantation, hepatic failure, active malignancy, receiving immunosuppressive therapy, and being infected by Human Immunodeficiency Virus (HIV) were significant factors.

In the third and fourth sections, all clinical and paraclinical presentations of COVID-19 associated with the poor prognosis of the COVID-19 were included. Clinical presentations included clinicians' overall assessment of the COVID-19 prognosis based on the frailty scale (14), hypoxia based on disparate levels of oxygen saturation (SpO_2_), hypotension and organ failure based on mean arterial pressure (MAP), the dosage of vasoactive agents required, having disseminated intravascular coagulation (DIC) and cardiac arrest. Paraclinical presentation of patients was included leukopenia, lymphopenia, low platelet counts, high bilirubin, creatinine, lactate-dehydrogenase (LDH), troponin, erythrocyte sedimentation rate (ESR), C-reactive protein (CPR), ferritin, and D-dimer.

### Data Analysis

To quantify the opinions of the Delphi panel, we asked the participants to rate each prioritising factor based on three scores; “−1” was considered as low priority, “+1” high priority, and “Zero” which indicated that the factor should not be deciding at all and was considered “equal priority.” The central tendency statistics, including mean, median, and mode, in addition to 95% confidence intervals (95% CI), standard deviation (SD), interquartile ranges (IQR), and skewness, were reported. The Mann-Whitney U test and Kruskal-Wallis one-way analysis of variance tests were applied to define the differences between the means of two groups and three groups or more, respectively. Statistical analyses were performed using IBM SPSS Statistics 21. A probability level of <0.05 was considered significant.

## Results

Of 38 participants, responses of 35 (92.1%) participants were analysed. The mean (SD) age of participants was 50.1 (9.0), range 39–78, being 51.2 (9.8) among men and 47.2 (5.4) among women. Among participants, 22 (64.7%) declared that they had not encountered the situation of deciding to allocate invasive mechanical ventilators in the setting of scarcity during the COVID-19 pandemic. Other sociodemographic characteristics of participants are presented in Table 1.

**Table 1:** Sociodemographic characteristics of participants.

**Variable**	***n* (%)**
**Sex**	
Female	9 (25.7)
Male	26 (74.3)
**Country**	
Iran	14 (40.0)
Belgium	3 (8.6)
United States of America	3 (8.6)
United Kingdom	2 (5.7)
India	2 (5.7)
Norway	2 (5.7)
Other	9 (25.7)
**Specialty**	
Intensive care medicine	15 (42.9)
Internal medicine	7 (20.0)
Emergency medicine	7 (20.0)
Infectious diseases	4 (11.4)
Forensic medicine	2 (5.7)

Among various non-medical prioritising determinants, most participants believed that younger ages, healthcare workers, and the mothers of children under 5 years should be considered a high priority in allocating mechanical ventilators. As many as 26 (74.3%) participants reported high priority for patients under 80 years of age; however, 16 (45.7%) participants said they would give lower priority to patients aged 80 or more. There was no consensus regarding the prioritisation of ventilator allocation for patients' smoking and drug abuse status (Table 2).

**Table 2:** Responses on prioritising ventilator allocation regarding non-medical characteristics of patients.

**Factor**	**Low priority (%)**	**Equal priority (%)**	**High priority (%)**	**Mean (SD)**	**95% CI**	**Median (IQR)**	**Skewness**
**Age**							
<19 years	2 (5.7)	7 (20.0)	26 (74.3)	0.7 (0.6)	0.5, 0.9	1 (0, 1)	−1.7
20–49	1 (2.9)	6 (17.1)	28 (80.0)	0.8 (0.5)	0.6, 0.9	1 (1, 1)	−2.0
50–59	0 (0.0)	5 (14.3)	30 (85.7)	0.8 (0.4)	0.7, 1.0	1 (1, 1)	−2.0
60–69	0 (0.0)	6 (17.1)	29 (82.9)	0.8 (0.4)	0.6, 0.9	1 (1, 1)	−1.5
70–74	2 (5.7)	10 (28.7)	23 (65.6)	0.6 (0.6)	0.3, 0.7	1 (0, 1)	−1.2
75–79	8 (22.8)	10 (28.6)	17 (48.6)	0.3 (0.8)	0.0, 0.5	1 (0, 1)	−0.5
80–84	14 (40.0)	6 (17.1)	15 (42.9)	0.0 (0.9)	−0.4, 0.3	1 (−1, 1)	−0.1
≥85 years	17 (48.6)	5 (14.3)	13 (37.1)	−0.1 (0.9)	−0.4, 0.2	−1 (−1, 1)	0.2
**Occupation**							
Frontline HCW^*^	1 (2.9)	11 (31.4)	23 (65.7)	0.6 (0.5)	0.4, 0.8	1 (0, 1)	−1.0
Non-frontline HCW	1 (2.9)	14 (40.0)	20 (57.1)	0.5 (0.6)	0.3, 0.7	1 (0, 1)	−0.6
Smoking	10 (28.6)	15 (42.8)	10 (28.6)	0.3 (0.6)	−0.3, 0.3	0 (−1, 1)	−0.3
Drug abuse	14 (40.0)	16 (45.7)	5 (14.3)	−0.3 (0.7)	−0.5, 0.0	0 (−1, 0)	0.4
Mother of child under 5 years	1 (2.9)	7 (20.0)	27 (77.1)	0.6 (0.6)	0.5, 0.9	1 (0, 1)	−1.6

* *Healthcare worker*.

When the Delphi panel was asked to prioritise patients regarding their underlying health condition, 31 (88.6%) participants recommended that pregnant women take high priority. Among underlying diseases, 27 (77.1%) and 25 (71.4%) participants reported low priority for active malignancy and neurodegenerative diseases, respectively. Although patients with BMI>40, diabetes mellitus, chronic kidney disease, and chronic respiratory failure received low priority, the number of participants reporting high or equal priority for the diseases was high as well. The participants' responses on prioritising ventilator allocation regarding underlying health conditions are presented in Table 3.

**Table 3:** Responses on prioritising ventilator allocation regarding the underlying health condition of patients.

**Factor**	**Low priority (%)**	**Equal priority (%)**	**High priority (%)**	**Mean (SD)**	**95% CI**	**Median (IQR)**	**Skewness**
**Obesity**							
30 < BMI ≤35	2 (5.7)	19 (54.3)	14 (40.0)	0.3 (0.6)	0.1, 0.6	0 (0, 1)	−0.3
35 < BMI ≤40	8 (22.9)	18 (51.4)	9 (25.7)	0.0 (0.7)	−0.2, 0.3	0 (0, 1)	0.0
BMI >40	18 (51.4)	10 (28.6)	7 (20.0)	−0.3 (0.8)	−0.6, 0.0	−1 (−1, 0)	0.6
Pregnancy	0 (0.0)	4 (11.4)	31 (88.6)	0.9 (0.3)	0.7, 1.0	1 (1, 1)	−2.3
Uncontrolled hypertension	8 (22.9)	16 (45.7)	11 (31.4)	0.1 (0.7)	−0.3, 0.4	0 (−1, 1)	−0.1
Ischemic heart disease	10 (28.6)	15 (42.8)	10 (28.6)	0.0 (0.8)	−0.3, 0.3	0 (−1, 1)	0.0
Diabetes mellitus	13 (37.1)	9 (25.8)	13 (37.1)	0.0 (0.9)	−0.3, 0.3	0 (−1, 1)	0.0
Chronic kidney disease	13 (37.1)	12 (34.3)	10 (28.6)	−0.1 (0.8)	−0.4, 0.2	0 (−1, 1)	0.2
Neurodegenerative diseases	25 (71.4)	5 (14.3)	5 (14.3)	−0.5 (0.8)	−0.8, −0.3	−1 (−1, 0)	1.1
Chronic respiratory failure	20 (57.2)	4 (11.4)	11 (31.4)	−0.3 (0.9)	−0.6, 0.1	−1 (−1, 1)	0.6
Organ transplantation	6 (17.1)	14 (40.0)	15 (42.9)	0.3 (0.7)	0.0, 0.5	0 (0, 1)	−0.5
Immunosuppressive use	7 (20.0)	15 (42.9)	13 (37.1)	0.2 (0.8)	−0.1, 0.4	0 (0, 1)	−0.3
HIV/AIDS	10 (28.6)	19 (54.3)	6 (17.1)	−0.1 (0.7)	−0.4, 0.1	0 (−1, 0)	0.1
Hepatic failure	20 (57.2)	6 (17.1)	9 (25.7)	−0.3 (0.9)	−0.6, 0.0	−1 (−1, 1)	0.7
Active malignancy	27 (77.1)	3 (8.6)	5 (14.3)	−0.6 (0.7)	−0.9, −0.4	−1 (−1, −1)	1.7

While the Delphi panel assigned a high priority for hypotensive and hypoxic patients, the end stages of hypotension, including DIC and cardiac arrest, received lower priority. Considering the clinician's judgment about the prognosis of COVID-19 based on the frailty scale, severely frail and terminally ill patients were given lower priority than very fit, well, and managing well patients (Table 4).

**Table 4:** Responses on prioritising ventilator allocation regarding clinical presentation of COVID-19.

**Factor**	**Low priority (%)**	**Equal priority (%)**	**High priority (%)**	**Mean (SD)**	**95% CI**	**Median (IQR)**	**Skewness**
**Frailty scale**							
Very fit	2 (5.7)	8 (22.9)	25 (71.4)	0.7 (0.6)	0.5, 0.9	1 (0, 1)	−1.6
Well	3 (8.6)	7 (20.0)	25 (71.4)	0.6 (0.6)	0.4, 0.9	1 (0, 1)	−1.6
Managing well	1 (2.9)	11 (31.4)	23 (65.7)	0.6 (0.5)	0.4, 0.8	1 (0, 1)	−1.1
Vulnerable	2 (5.7)	12 (34.3)	21 (60.0)	0.5 (0.6)	0.3, 0.8	1 (0, 1)	−1.0
Mildly frail	6 (17.1)	10 (28.6)	19 (54.3)	0.4 (0.8)	0.1, 0.6	1 (0, 1)	−0.8
Moderately frail	11 (31.4)	12 (34.3)	12 (34.3)	0.0 (0.8)	−0.3, 0.3	0 (−1, 1)	−0.1
Severely frail	22 (62.8)	3 (8.6)	10 (28.6)	−0.3 (0.9)	−0.7, 0.0	−1 (−1, 1)	0.8
Very severely frail	23 (65.7)	3 (8.6)	9 (25.7)	−0.4 (0.9)	−0.7, −0.1	−1 (−1, 1)	0.9
Terminally Ill	28 (80.0)	2 (5.7)	5 (14.3)	−0.7 (0.7)	−0.9, −0.4	−1 (−1, −1)	1.8
**Hypoxia**							
88 < SpO2 ≤ 93%	4 (11.4)	9 (25.8)	22 (62.8)	0.5 (0.7)	0.3, 0.8	1 (0, 1)	−1.0
SpO2 <88%	3 (8.6)	6 (17.1)	26 (74.3)	0.7 (0.6)	0.4, 0.9	1 (0, 1)	−1.7
**Hypotension and organ failure**							
MAP^*^ <70 mmHg	2 (5.7)	12 (34.3)	21 (60.0)	0.5 (0.6)	0.3, 0.8	1 (0, 1)	−1.0
Dopamine ≤5 or Dobutamine (any dose)	2 (5.7)	13 (37.1)	20 (57.2)	0.5 (0.6)	0.3, 0.7	1 (0, 1)	−0.9
Dopamine >5, Epinephrine ≤0.1, or norepinephrine ≤0.1	4 (11.4)	12 (34.3)	19 (54.3)	0.4 (0.7)	0.2, 0.7	1 (0, 1)	−0.8
Dopamine >15, Epinephrine >0.1, or norepinephrine >0.1	7 (20.0)	11 (31.4)	17 (48.6)	0.3 (0.8)	0.0, 0.6	0 (0, 1)	−0.6
DIC^**^	13 (37.2)	11 (31.4)	11 (31.4)	0.0 (0.9)	−0.3, 0.3	0 (−1, 1)	0.7
Cardiac arrest	28 (80.0)	2 (5.7)	5 (14.3)	−0.7 (0.7)	−1, −0.4	−1 (−1, −1)	0.5

* * Mean Arterial Pressure*.

** *Disseminated intravascular coagulation*.

Considering the importance of paraclinical factors in anticipating the prognosis of COVID-19 among patients, we asked the panellists to rate every paraclinical factor, including blood cell counts, liver and kidney function tests, inflammatory factors, troponin, and D-dimer tests. The majority of paraclinical factors associated with the severity of COVID-19 were considered unimportant in resource allocation by panellists (Table 5).

**Table 5:** Responses on prioritising ventilator allocation regarding laboratory presentation of COVID-19.

**Factor**	**Low priority (%)**	**Equal priority (%)**	**High priority (%)**	**Mean (SD)**	**95% CI**	**Median (IQR)**	**Skewness**
Leukopenia	5 (14.2)	20 (57.2)	10 (28.6)	0.2 (0.7)	0.0, 0.5	0 (0, 1)	−0.3
Lymphopenia	4 (11.4)	16 (45.7)	15 (42.9)	0.3 (0.7)	0.1, 0.6	0 (0, 1)	−0.5
**Low platelet count**							
100 < PLT ≤149	1 (2.9)	27 (77.1)	7 (20.0)	0.2 (0.5)	0.1, 0.4	0 (0, 1)	0.6
50 < PLT ≤99	2 (5.7)	20 (57.2)	13 (37.1)	0.3 (0.6)	0.1, 0.5	0 (0, 1)	−0.2
20 < PLT ≤49	9 (25.7)	15 (42.9)	11 (31.4)	0.1 (0.8)	−0.2, 0.3	0 (−1, 1)	−0.1
PLT <20	11 (31.4)	15 (42.9)	9 (25.7)	−0.1 (0.8)	−0.3, 0.2	0 (−1, 1)	0.1
High LDH	3 (8.6)	21 (60.0)	11 (31.4)	0.3 (0.6)	0.1, 0.5	0 (0, 1)	−0.2
High troponin	6 (17.1)	16 (45.7)	13 (37.2)	0.2 (0.7)	0.0, 0.5	0 (0, 1)	−0.4
**High bilirubin**							
1.2–1.9 mg/dL	4 (11.4)	25 (71.5)	6 (17.1)	0.1 (0.6)	0.0, 0.3	0 (0, 0)	0.0
2.0–5.9 mg/dL	4 (11.4)	22 (62.9)	9 (25.7)	0.1 (0.6)	−0.1, 0.4	0 (0, 1)	−0.1
6.0–11.9 mg/dL	14 (40.0)	13 (37.1)	8 (22.9)	−0.2 (0.8)	−0.5, 0.1	0 (−1, 0)	0.3
≥12.0 mg/dL	16 (45.7)	10 (28.6)	9 (25.7)	−0.2 (0.8)	−0.5, 0.1	0 (−1, 1)	0.4
**High creatinine**							
1.2–1.9 mg/dL	0 (0.0)	24 (68.6)	11 (31.4)	0.3 (0.5)	0.2, 0.5	0 (0, 1)	0.6
2.0–3.4 mg/dL	3 (8.6)	21 (60.0)	11 (31.4)	0.2 (0.6)	0.0, 0.4	0 (0, 1)	−0.1
3.5–4.9 mg/dL	7 (20.0)	18 (51.4)	10 (28.6)	0.1 (0.7)	−0.2, 0.3	0 (0, 1)	−0.1
≥5.0 mg/dL	10 (28.6)	14 (40.0)	11 (31.4)	0.0 (0.8)	−0.2, 0.3	0 (−1, 1)	−0.1
High ESR	2 (5.7)	24 (68.6)	9 (25.7)	0.2 (0.5)	0.0, 0.4	0 (0, 1)	0.2
High CRP	2 (5.7)	20 (57.2)	13 (37.1)	0.3 (0.6)	0.1, 0.5	0 (0, 1)	−1.5
High D-dimer	1 (2.9)	21 (60.0)	13 (37.1)	0.3 (0.5)	0.2, 0.5	0 (0, 1)	0.1
High ferritin	2 (5.7)	25 (71.4)	8 (22.9)	0.2 (0.5)	0.0, 0.4	0 (0, 0)	0.3

No correlations were observed with participants' responses on resources allocation and their age, religion, country of residence, the field of study, and the dilemma of allocating invasive mechanical ventilators in the setting of scarcity during the COVID-19 pandemic.

## Discussion

The study showed that the panel considered younger age groups, healthcare workers, and mothers of children under 5 years for prioritising mechanical ventilators. While most participants reported high priority for patients under 80 years of age, almost half of participants said they would give lower priority to patients aged 80 or more. There was no consensus regarding the prioritisation of ventilator allocation for patients' smoking and drug abuse status.

Some 89% of participants recommended that pregnant women must take high priority. Many guidelines also prioritised pregnant women, younger age groups, and healthcare professionals to allocate ventilators (15). There is evidence that the mortality due to COVID-19 is lower among younger age groups. Nevertheless, young hospitalised patients with COVID-19 regularly require ventilators for extended periods (16). Thus, cohort and investigational studies could help healthcare professionals and experts.

Although patients with BMI>40, diabetes mellitus, chronic kidney disease, and chronic respiratory failure received low priority, the number of participants reporting high or equal priority for the diseases was high as well. While the panel considered a high priority for hypotensive and hypoxic patients, the end stages of hypotension, including DIC and cardiac arrest, received lower priority.

More than two-thirds of participants considered patients with active malignancy and neurodegenerative diseases to have low priority. COVID-19 pandemic has disrupted the conventional care delivery for both malignancies and neurodegenerative diseases (17, 18). Patients with neurodegenerative diseases, which is more common among advanced age groups (19), often live in residential homes, which puts them at greater risk of COVID-19 transmission (20). Similar to considering these groups as low-priority in our study, many resource allocation guidelines have excluded these patients (21), which could put them at risk of systemic discrimination in the near future (22).

Among underlying diseases, COPD is reported to be an independent risk factor for all-cause mortality among patients with COVID-19 (23). Hypertension and uncontrolled hypertension were the most common comorbidity among hospitalised patients with COVID-19 infection (24).

The majority of paraclinical factors associated with the severity of COVID-19 were considered unimportant in resource allocation. There is evidence that LDH and CRP independently predicted ventilation requirements among COVID-19 patients (16).

Considering the clinician's judgment about the prognosis of COVID-19 based on the frailty scale, severely frail and terminally ill patients were given lower priority than very fit, well, and managing well patients. Evidence shows that the frailty scale is linearly associated with increased mortality due to COVID-19 (25). Although some studies proposed using Sequential Organ Failure Assessment (SOFA) score for prioritising ventilator allocation in the early days of the pandemic (26), the SOFA score has been shown to have inadequate accuracy for ventilator triage of patients with COVID-19 (27). The combination of the frailty scale with the SOFA score did not improve the performance of the SOFA score either (28). Thus, better alternatives are needed for prognostic prediction of patients with COVID-19 pneumonia requiring mechanical ventilation.

Unresolved ethical dilemmas regarding the fair allocation of ventilators threaten the success of the response to a public health emergency. Nevertheless, not all healthcare systems have developed allocation guidelines (26, 29, 30). Some studies challenge the “save the most lives” strategy. A study proposes that the following considerations be taken into account, when necessary while allocating scarce resources: maximising survival to hospital discharge, maximising the number of life-years saved, maximising individuals' chances to live through each of life's stages, the severity of impairment, and patients' instrumental value into prioritisation considerations. In this sense, the public also needs to participate in choosing among ethically permissible allocation strategies (7, 31). Some studies have investigated people's opinions on the fair allocation of ventilators (32). A community-based survey reported that people considered age, expected ventilation effectiveness, smoking status, having dependents, being a healthcare worker, and having disabilities to be of importance in resource allocation (33).

## Strengths and Limitations

This is among the few studies investigating the experts' opinions on priorities towards fair allocation of mechanical ventilators during the COVID-19 pandemic. Findings could empower public health authorities better to understand experts' opinions to be considered in future guidelines. It is worth mentioning that the COVID-19 pandemic disrupted the supply chain of medical resources, which was only successful when demand was predictable (34). Focusing on the fair allocation of ventilators during this crisis should not distract the authorities from optimising the supply chain.

We realise the limitations of the study. The number of participants was limited; however, the pandemic disrupted people's daily schedule worldwide, and experts were no exception (35). Nevertheless, given the response rate, the generalisation of results could be limited. Considering that the panel was approached based on the acquaintance of authors, our sample was over-representative of colleagues in the authors' network. Using the authors' network would increase the chance of the panel's participation in the study, given that experts would be too busy during the pandemic and would probably ignore emails from unfamiliar senders. While the study had a poor representation of some regions, especially considering different social, religious and healthcare systems approaches, the findings could be used as a basis for a broader represented experts' panel.

## Conclusion

The panel considered younger age groups, healthcare workers, pregnant mothers, and mothers of children under 5 years for prioritising mechanical ventilators. There was no general consensus regarding the prioritisation of ventilator allocation based on the patient's laboratory profile, underlying diseases, or drug abuse. It could be suggested that more research is essential to develop comprehensive resource allocation strategies which are easy to apply, objective, accurate, reproducible, and would not discriminate against vulnerable populations.

## Data Availability Statement

The raw data supporting the conclusions of this article will be made available by the authors, without undue reservation.

## Ethics Statement

The studies involving human participants were reviewed and approved by Ethical Committee of Shahid Beheshti University of Medical Sciences, Tehran, Iran under the reference code IR.SBMU.RETECH.REC.1399.103. Written informed consent for participation was not required for this study in accordance with the national legislation and the institutional requirements.

## Author Contributions

A-AK, NZ, HH-M, S-HG, and MA-K: study conception and design. A-AK, S-HG, and MA-K: acquisition, analysis, or interpretation of data. S-HG and MA-K: drafting of the manuscript. A-AK, HH-M, and NZ: critical revision of the manuscript for important intellectual content. A-AK: study supervision. The corresponding authors attests that all listed authors meet the authorship criteria and that no others meeting the criteria have been omitted. All authors contributed to the article and approved the submitted version.

## Funding

This study was supported by Social Determinants of Health Research Center, Shahid Beheshti University of Medical Sciences, Tehran, Iran under code 23526. The funding body was not in any ways involved in the design of the study and collection, analysis, and interpretation of data and in writing the manuscript.

## Conflict of Interest

The authors declare that the research was conducted in the absence of any commercial or financial relationships that could be construed as a potential conflict of interest.

## Publisher's Note

All claims expressed in this article are solely those of the authors and do not necessarily represent those of their affiliated organizations, or those of the publisher, the editors and the reviewers. Any product that may be evaluated in this article, or claim that may be made by its manufacturer, is not guaranteed or endorsed by the publisher.
